# Essential Oil Profile and Yield of Corolla, Calyx, Leaf, and Whole Flowering Top of Cultivated *Lavandula angustifolia* Mill. (Lamiaceae) from Utah

**DOI:** 10.3390/molecules26082343

**Published:** 2021-04-17

**Authors:** Tyler M. Wilson, Ariel Poulson, Chris Packer, Richard E. Carlson, R. Michael Buch

**Affiliations:** D. Gary Young Research Institute, Lehi, UT 84043, USA; apoulson@youngliving.com (A.P.); cpacker@youngliving.com (C.P.); richcarlson@youngliving.com (R.E.C.); mbuch@youngliving.com (R.M.B.)

**Keywords:** *Lavandula angustifolia*, Lamiaceae, essential oil, Utah, corolla, calyx, leaf, yield

## Abstract

*Lavandula angustifolia* Mill. (lavender) is an essential-oil-bearing plant in the Lamiaceae family. Volatile oil produced through the steam distillation of lavender was examined to establish the essential oil yield and aromatic profile from each portion of the plant—namely, the corolla, calyx, leaf, and whole flowering top. The resulting essential oils were analyzed by GC-FID and GC-MS. The different plant parts generally shared similar compounds but in varying relative percentages. Aromatic profiles of the whole flowering top and calyx were similar, with prominent compounds being linalool acetate (34.3%, 32.0%), linalool (26.5%, 32.9%), lavandulyl acetate (5.6%, 4.9%), terpinen-4-ol (5.3%, 7.0%), and (*Z*)-β-ocimene (4.5%, 5.4%), respectively. Aromatic profiles for the corolla and leaf were unique. Prominent aromatic compounds of the corolla included linalool acetate (18.4%), linalool (10.8%), epi-α-cadinol (10.0%), borneol (7.3%), and lavandulyl acetate (6.3%). Prominent aromatic compounds of the leaf included epi-α-cadinol (19.8%), γ-cadinene (11.0%), borneol (6.0%), caryophyllene oxide (4.9%), and bornyl acetate (4.8%). Complete profiles and essential oil yields of corolla, calyx, leaf, and whole flowering top were established. This study establishes the influence the corolla, calyx, and leaf exert on the aromatic profile of the whole flowering top and provides insight into authentication of lavender essential oil.

## 1. Introduction

Lavender, *Lavandula angustifolia* Mill., is an aromatic shrub in the Lamiaceae family that is native to the Mediterranean and cultivated throughout the world [1,2]. Many plants in the Lamiaceae family are aromatic and contain different types of essential-oil-bearing glands [3]. *Lavandula* species, including *L. angustifolia*, contain both capitate and peltate glandular trichomes [4,5] that must be ruptured or otherwise damaged to extract the essential oil [6].

Among the many traditional uses for the lavender plant, those commonly cited are the purported sedative and anti-inflammatory properties [2]. These traditional uses have been substantiated through recent studies [7,8,9,10]. Lavender essential oil has also been shown to have a positive effect on anxiety and depression in humans and in animal models [11,12,13,14,15]. Its pleasant aroma combined with its purported health benefits make lavender essential oil a popular choice for aromatherapy and a common ingredient in cosmetics, flavors, and fragrances. 

With such widespread use of lavender essential oil, adulteration is a concern. Several analytical methods have been developed to authenticate this essential oil [16,17,18]. A better understanding of the complete aromatic profile of genuine lavender would help to determine authenticity. 

The International Organization for Standardization (ISO) standards for lavender [17], lavandin Grosso (*Lavandula angustifolia* Mill. x *Lavandula latifolia* Medik.) [19], and spike lavender (*Lavandula latifolia* Medik.) [20] provide characteristics to assess the quality of each essential oil. The latter two essential oils can be used as adulterants of authentic lavender essential oil since both contain similar aromatic profiles to lavender [2,17,19,20,21]. The lavender ISO standard [17] states that prominent or otherwise important volatile compounds of lavender essential oil cultivated in the United States (interpreted as “other origins” of lavender) include limonene (nd–1%), 1,8-cineole (nd–3%), β-phellandrene (nd–1%), (*Z*)-β-ocimene (1–10%), (*E*)-β-ocimene (0.5–6%), 3-octanone (nd–3%), camphor (nd–1.5%), linalool (20–43%), linalool acetate (25–47%), lavandulol (nd–3%), terpinen-4-ol (nd–8%), lavandulyl acetate (nd–8%), and α-terpineol (nd–2%). Ranges for said compounds vary due to both geographic location and lavender cultivar [17,22,23,24]. Many other factors have also been shown to influence the yield and volatile profile of lavender essential oil, including the developmental stage of the flower [25], weather conditions [25], time of harvest [26], drying and storing conditions of plant material [26,27,28], distillation time [29], and the distillation or extraction technique employed [30,31,32,33].

The plant part used for extraction is an additional factor that affects both the yield and the volatile profile of lavender essential oil. Previous studies have shown that linalool, linalool acetate, lavandulyl acetate, and α-terpineol are the predominant constituents in the essential oil extracted from the flowering top, and yield was significantly higher in essential oil extracted from the flowering top compared to the stem [34,35]. Essential oil extracted from the leafy stem is prominent in borneol, (*E*)-caryophyllene, camphor, 1,8-cineole, and linalool [34,36]. The calyx is the main site of essential oil accumulation, followed by the leaves, stem, and corolla [37]. The flowering top, including the corolla and calyx complex, accumulates 10× the essential oil content as compared to the leaves, and the calyx accumulates 50× more when compared to the corolla [37]. To the authors’ knowledge, the steam-distilled essential oils of the corolla and calyx have never been fully examined. In this study, the essential oil profiles of *Lavandula angustifolia* corolla, calyx, leaf, and flowering top (including corolla, calyx, stem, and leaf) cultivated in Utah, USA are established and compared. Distillation yields of the various plant parts are also reported. Results provide further insight into cultivation and harvesting practices, and into the authentication of lavender. 

## 2. Results

**Figure 1:**
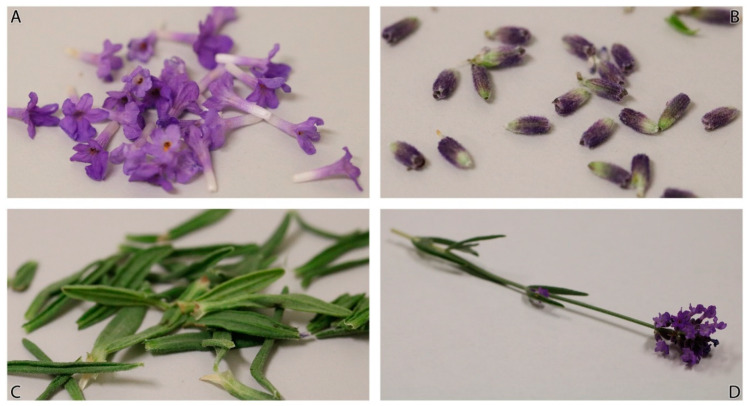
Portions of *Lavandula angustifolia* used: (**A**) corolla, (**B**) calyx, (**C**) leaf, (**D**) flowering top.

Consistent with previous findings [37], inflorescences contain flowers that are not synchronized in their development. The developmental stage of lavender and the portion of the plant that is harvested impact the aromatic profile and essential oil quality of lavender (Figure 1). 

The aromatic profiles of the corolla, calyx, leaf, and whole flowering top of *L. angustifolia* are detailed in Table 1. Each reported value is an average from three samples distilled from that portion of the plant. Essential oil samples were analyzed in triplicate to ensure reproducibility (SD < 1 for all compounds). Yield is detailed in Table 2. Consistent with previous findings [37], the calyx is the main site of essential oil accumulation (yield 1.3%). However, this study found that the corolla had the next highest yield (0.1%), followed by the leaf (0.05%). The whole flowering top, composed of corolla, calyx, stem, and leaf, had a yield of 0.7%.

Aromatic profiles of the whole flowering top and calyx were similar, with prominent compounds being linalool acetate (34.3%, 32.0%), linalool (26.5%, 32.9%), lavandulyl acetate (5.6%, 4.9%), terpinen-4-ol (5.3%, 7.0%), (*Z*)-β-ocimene (4.5%, 5.4%), and (*E*)-β-ocimene (2.9%, 1.6%). Both aromatic profiles, that of the whole flowering top and calyx, conform to the ranges established by the lavender ISO standard, showing that the composition of lavender essential oil is greatly influenced by the essential oils present in the calyx [17].

**Figure 2:**
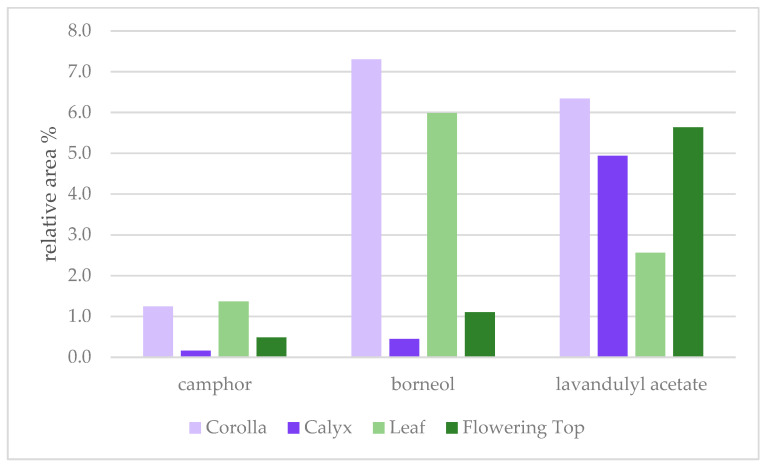
Relative area % of camphor, borneol, and lavandulyl acetate in each portion of *L. angustifolia*, namely the corolla, calyx, leaf, and whole flowering top. The profiles of the calyx and whole flowering top are similar. Despite the low yield of the corolla and leaf, both portions impact the profile of the whole flowering top.

Prominent aromatic compounds of the corolla include linalool acetate (18.4%), linalool (10.8%), epi-α-cadinol (10.0%), borneol (7.3%), and lavandulyl acetate (6.3%). Prominent aromatic compounds of the leaf include epi-α-cadinol (19.8%), γ-cadinene (11.0%), borneol (6.0%), caryophyllene oxide (4.9%), and bornyl acetate (4.8%). Several sesquiterpenoids are prominent in both the corolla and leaf, and show a decreasing trend from leaf, corolla, and whole flowering top, to calyx: γ-cadinene (11.0%, 5.1%, 0.7%, 0.4%), caryophyllene oxide (4.9%, 4.9%, 0.3%, 0.1%), and epi-α-cadinol (19.8%, 10.0%, 1.3%, 1.1%), respectively. While the yield of both corolla and leaf are substantially lower than that of the calyx, the unique profiles of both portions contribute to the overall profile of the flowering top. For instance, relative amounts of camphor, borneol, and lavandulyl acetate in the flowering top are influenced by both the corolla and leaf (Figure 2). Further, elevated levels of camphor, which is a particular concern with the adulteration of authentic lavender, could be unintentionally elevated by harvesting an abundance of leafy material.

## 3. Discussion

This study confirms that the calyx is the main site of essential oil accumulation and establishes, for the first time, the complete aromatic profile of the corolla and calyx. The calyx has the highest yield, followed by the whole flowering top, corolla, and leaf. The calyx and whole flowering top have similar aromatic profiles, with prominent compounds being linalool acetate, linalool, lavandulyl acetate, terpinen-4-ol, (*Z*)-β-ocimene, and (*E*)-β-ocimene. Interestingly, the profiles of both the calyx and whole flowering top are consistent with the lavender ISO standard ranges as previously defined under cultivated lavender from the United States. However, neither profile for the corolla or leaf alone are consistent with the lavender ISO standard ranges [17]. Linalool acetate, linalool, and lavandulyl acetate are prominent in the corolla, as are borneol and the sesquiterpenoid epi-α-cadinol. Prominent compounds in the leaf essential oil include epi-α-cadinol, γ-cadinene, borneol, caryophyllene oxide, and bornyl acetate. 

This study also establishes the influence exerted by the corolla and leaf, through both yield and unique profile, on the aromatic profile of the whole flowering top. While previous studies established both the qualitative and quantitative contribution of various plant parts to the essential oil of lavender [34,35,36,37], this is the first study to establish the complete profile and yield of each isolated plant part in relation to the complete flowering top. The yield of both the corolla and leaf are substantially lower than that of the calyx, yet several prominent compounds in both oils (i.e., 1,8-cineole, camphor, borneol, lavandulyl acetate, γ-cadinene, caryophyllene oxide, epi-α-cadinol) contribute to the overall profile of the flowering top. 

Understanding the contribution of each plant part to the essential oil of lavender provides insight into proper cultivation and harvesting practices, as well as a better understanding of authentication of natural lavender essential oil. Authentic lavender essential oil has low levels of camphor, typically lower than the upper limit established by the lavender ISO standard [17,22,24,28,30,31,35]. Elevated levels of camphor are an indicator of the addition of lavandin Grosso and/or spike lavender, which have camphor levels of 6–8.5% and 8–16%, respectively [2,19,20,21]. When determining the quality and authenticity of lavender essential oil, distinguishing the cause of elevated camphor levels between natural (corolla and/or leaf from lavender) and unnatural origin (addition of spike lavender and/or lavandin Grosso) is important. Factors such as the developmental stage of the inflorescences and the amount of leaf material harvested impact the overall aromatic profile of lavender essential oil. 

The current study was done using plant material collected during a single week. Future research will focus on the contribution (qualitative and quantitative) each plant part provides during the entirety of the lavender harvest. 

## 4. Materials and Methods

*Lavandula angustifolia* plant material was collected during the second week of July 2020 from cultivated fields in Juab County, Utah, USA (39°52′18′′N 111°50′46′′W; 1503 m elevation). Flowering tops were cut from mature (4–6-year-old) plants (*n* = 10) and were meticulously divided into four groups, namely the corolla, calyx, leaf, and whole flowering top (comprised of corolla, calyx, leaf, stem) to determine the weight, yield, and aromatic profile of each portion of the plant (Figure 1). Representative voucher samples are held in the Utah Valley University Herbarium (UVSC): *L. angustifolia* Mill., Wilson 2020-01, -02, -03 (UVSC).

Plant material was prepared for laboratory-scale distillation as follows: corolla, calyx, leaf, and whole flowering top were separated, bagged, and stored at −20 ± 2 °C until steam distilled. Steam distillation was performed in triplicate, resulting in 3 distillations per plant portion and 12 distillations over the course of this project.

Laboratory-scale distillation was as follows: 1.5 L of water added to 2 L steam generator that fed to a 2 L distillation chamber, plant material accurately weighed and added to the distillation chamber, distillation for 1.5 h from pass-over by indirect steam, essential oil separated by a cooled condenser and Florentine flask. Essential oil samples were each filtered and stored at room temperature in a sealed amber glass bottle until analysis.

Essential oils were analyzed, and volatile compounds identified, by GC/MS using an Agilent 7890B GC/5977B MSD and J&W DB-5, 0.25 mm × 60 m, 0.25 μm film thickness, fused silica capillary column. Operating conditions: 0.1 μL of sample (20% soln. for essential oils in hexane), 150:1 split ratio, initial oven temperature of 40 °C with an initial hold time of 5 min, oven ramp rate of 4.5 °C per minute to 310 °C with a hold time of 5 min. The electron ionization energy was 70 eV, scan range 35–650 amu, scan rate 2.4 scans per second, source temperature 230 °C, and quadrupole temperature 150 °C. Volatile compounds were identified using the Adams volatile oil library [38] using Chemstation library search in conjunction with retention indices. Note that limonene/β-phellandrene, lavandulol/borneol, and α-trans-bergamotene/coumarin elute as single peaks. Their amounts were determined by the ratio of masses 68 and 79 (limonene), 77 and 93 (β-phellandrene), 69 and 111 (lavandulol), 95 and 110 (borneol), 93 and 119 (α-trans-bergamotene), and 118 and 146 (coumarin). Volatile compounds were quantified and are reported as a relative area percent by GC-FID using an Agilent 7890B and J&W DB-5, 0.25 mm × 60 m, 0.25 μm film thickness, fused silica capillary column. Operating conditions: 0.1 μL of sample (20% soln. for essential oils in hexane, 1% for reference compounds in hexane, 0.1% soln. for C7–C30 alkanes in hexane), 25:1 split injection, initial oven temperature at 40 °C with an initial hold time of 2 min, oven ramp rate of 3.0 °C per minute to 250 °C with a hold time of 3 min. Essential oil samples were analyzed in triplicate. Compounds were identified using retention indices coupled with the retention time data of reference compounds (MilliporeSigma, Sigma-Aldrich, St. Louis, MS, USA). 

The percent yield was calculated as the ratio of the mass of processed plant material immediately before distillation to the mass of essential oil produced, multiplied by 100.

## Figures and Tables

**Table 1:** Aromatic profile of *L. angustifolia* essential oil from the corolla, calyx, leaf, and whole flowering top. Each reported value below represents the average of three essential oil samples distilled from each portion. Each essential oil sample was analyzed in triplicate. Values less than 0.1% are denoted as trace (t) and those not detected in a portion of the plant as not detectable (nd). Unidentified compounds less than 0.5% are not included. KI is the Kovat’s Index value obtained using a linear calculation on DB-5 column [38]. Relative area percent was determined by GC-FID.

KI	Compound	Corolla	Calyx	Leaf	Flowering Top
921	tricyclene	nd	nd	t	0.1
924	α-thujene	nd	0.1	t	0.2
932	α-pinene	t	0.2	0.1	0.3
946	camphene	0.1	0.1	0.6	0.4
969	sabinene	t	t	0.1	0.1
974	1-octen-3-ol	0.1	0.2	nd	nd
974	β-pinene	nd	nd	0.2	0.2
979	3-octanone	0.1	0.7	0.2	1.1
988	myrcene	0.1	0.4	0.6	0.6
988	3-octanol	t	nd	nd	nd
993	butyl butanoate	nd	0.2	nd	0.2
1002	α-phellandrene	t	t	0.2	t
1007	hexyl acetate	t	0.3	nd	nd
1008	δ-3-carene	nd	nd	1.8	0.5
1014	α-terpinene	t	t	t	0.2
1020	p-cymene	t	t	0.7	0.1
1022	ο-cymene	0.2	0.1	1.5	0.4
1024	limonene	0.3	0.2	1.8	0.4
1025	β-phellandrene	t	0.1	2.3	0.3
1026	1,8-cineole	0.9	0.3	1.2	1.5
1032	(*Z*)-β-ocimene	0.5	5.4	0.3	4.5
1044	(*E*)-β-ocimene	0.3	1.6	0.2	2.9
1054	γ-terpinene	t	0.2	0.1	0.1
1065	cis-sabinene hydrate	0.1	0.2	0.1	0.2
1067	cis-linalool oxide	t	0.1	nd	t
1084	trans-linalool oxide	t	t	nd	t
1085	p-mentha-2,4(8)-diene	nd	nd	0.3	nd
1086	terpinolene	t	t	0.2	t
1095	linalool	10.8	32.9	1.1	26.5
1100	n-nonanal	0.2	nd	nd	nd
1101	hexyl propanoate	nd	nd	nd	0.1
1110	1-octen-3-yl acetate	0.4	0.5	1.5	0.7
1118	cis-p-menth-2-en-1-ol	0.2	nd	0.1	nd
1120	3-octanol acetate	nd	0.1	nd	0.1
1122	α-campholenal	0.1	nd	0.1	nd
1128	allo-ocimene	nd	0.4	nd	0.3
1132	cis-limonene oxide	0.1	nd	t	nd
1135	trans-pinocarveol	0.2	nd	t	nd
1136	trans-p-menth-2-en-1-ol	0.1	nd	t	nd
1141	camphor	1.2	0.2	1.4	0.5
1145	camphene hydrate	nd	nd	t	nd
1155	isoborneol	0.1	nd	0.1	nd
1165	lavandulol	0.8	1.0	0.3	0.6
1165	borneol	7.3	0.4	6.0	1.1
1174	terpinen-4-ol	1.9	7.0	0.4	5.3
1179	p-cymen-8-ol	0.2	t	0.1	0.1
1183	cryptone	1.7	nd	0.3	0.2
1186	α-terpineol	0.3	0.4	0.1	0.3
1191	hexyl butanoate	nd	0.4	nd	0.4
1194	myrtenol	0.3	nd	0.2	t
1207	trans-piperitol	0.3	nd	0.1	nd
1215	trans-carveol	0.2	nd	0.1	nd
1227	nerol	nd	0.1	nd	t
1235	isobornyl formate	1.0	0.1	1.6	0.2
1238	cumin aldehyde	2.0	nd	1.3	0.2
1239	carvone	0.5	nd	0.3	t
1254	linalool acetate	18.4	32.0	3.0	34.3
1284	bornyl acetate	2.3	nd	4.8	nd
1288	lavandulyl acetate	6.3	4.9	2.6	5.6
1289	p-cymen-7-ol	0.5	nd	nd	t
1298	carvacrol	nd	nd	t	nd
1330	hexyl tiglate	t	t	nd	t
1330	3-oxo-p-menth-1-en-7-al	0.1	nd	nd	nd
1343	benzyl butanoate	nd	t	nd	nd
1359	neryl acetate	0.6	0.2	0.6	0.2
1379	geranyl acetate	0.9	0.4	2.5	0.3
1382	hexyl hexanoate	nd	0.1	nd	nd
1389	β-elemene	nd	nd	0.1	nd
1410	α-cedrene	0.1	nd	0.3	nd
1411	α-cis-bergamotene	nd	nd	0.1	nd
1416	α-santalene	0.8	0.2	1.1	0.2
1417	(*E*)-caryophyllene	1.7	2.5	2.2	2.3
1419	β-cedrene	t	nd	0.1	nd
1432	α-trans-bergamotene	0.2	0.1	0.3	0.1
1432	coumarin	nd	nd	0.5	0.2
1440	(*Z*)-β-farnesene	1.4	1.8	nd	1.6
1452	α-humulene	nd	0.1	0.1	0.1
1465	cis-muurola-4(14),5-diene	nd	0.1	0.8	0.1
1471	dauca-5,8-diene	0.1	nd	nd	nd
1474	10-epi-β-acoradiene	t	nd	0.2	nd
1480	germacrene D	0.3	0.4	0.3	0.3
1505	β-bisabolene	0.1	0.1	t	t
1513	γ-cadinene	5.1	0.4	11.0	0.7
1528	cis-calamenene	0.2	t	0.6	t
1537	α-cadinene	nd	nd	0.1	nd
1582	caryophyllene oxide	4.9	0.1	4.9	0.3
1627	1-epi-cubenol	1.0	0.1	1.8	0.1
1638	epi-α-cadinol	10.0	1.1	19.8	1.3
1685	germacra-4(15),5,10(14)-trien-1-α-ol	0.4	nd	nd	nd
1688	cis-14-nor-muurol-5-en-4-one	1.2	0.1	1.2	0.1
^1^ 1744	unknown compound	1.4	t	1.5	t
1759	benzyl benzoate	t	t	t	t
^1^ 1847	unknown compound	1.3	nd	0.2	nd

^1^ The KI was calculated using alkane standards.

**Table 2:** Distribution of mass and essential oil (EO) yield averaged from lavender samples.

		Mass Distilled (g)	Yield EO (g)	Yield EO (%)
Flowering Top	1	134.60	1.12	0.83
	2	129.48	0.86	0.66
	3	129.32	0.65	0.50
	Avg:	131.13	0.88	0.66
	%RSD (*n* = 3)			24.9
Corolla	1	48.44	0.04	0.08
	2	43.04	0.05	0.12
	3	50.57	0.05	0.10
	Avg:	47.35	0.05	0.10
	%RSD (*n* = 3)			20.0
Calyx	1	87.05	1.09	1.25
	2	107.02	1.42	1.33
	3	102.67	1.33	1.30
	Avg:	98.91	1.28	1.29
	%RSD (*n* = 3)			3.1
Leaf	1	118.53	0.06	0.05
	2	100.03	0.05	0.05
	3	123.36	0.06	0.05
	Avg:	113.97	0.06	0.05
	%RSD (*n* = 3)			0.0

## Data Availability

The data presented in this study are available upon request from the corresponding author.

## Sample Availability

Samples of the compounds are not available from the authors.
